# Clinical and Radiological Features of Perianal Fistula: An MRI-Based Study in Addis Ababa, Ethiopia

**DOI:** 10.4314/ejhs.v34i1.6S

**Published:** 2024-10

**Authors:** Semira Abrar Issa, Tesfaye Kebede Legesse, Assefa Getachew Kebede, Alemayehu Bedane Worke, Ashenafi Aberra Buser

**Affiliations:** 1 Addis Ababa University, College of Health Sciences, Medical Faculty, Department of Radiology, Addis Ababa, Ethiopia; 2 St. Paul Millennium Medical College, Department of Radiology, Addis Ababa, Ethiopia

**Keywords:** Perianal fistula, MRI, recurrence, St. James Classification, Parks Classification

## Abstract

**Background:**

Perianal fistula refers to an abnormal connection between the anal canal and the perianal skin or perineum. Magnetic Resonance Imaging (MRI) plays a crucial role in accurately characterizing perianal fistulas, which informs surgical strategies and helps minimize recurrence.

**Methods:**

This cross-sectional study was conducted at a single diagnostic imaging center in Addis Ababa, utilizing retrospectively collected data from May 2023 to June 2024. All patients referred for suspected perianal fistulas were included. MRI scans were reviewed by a radiologist in body imaging fellowship and a body imaging subspecialist. Findings, along with sociodemographic data, were documented in a structured questionnaire and analyzed using SPSS.

**Results:**

The study encompassed 304 primary fistula tracts in 276 patients, with 233 (84.4%) being male. Complex fistulas were identified in 83 patients (30.1%), 43 (15.6%) had secondary extensions, and 64 (27.1%) presented with abscess collections. The most common type of fistula, according to the Parks classification, was intersphincteric, observed in 263 cases (86.5%). The St. James University Hospital grades 1 and 2 were predominant, representing 176 (57.9%) and 62 (20.4%) of the cases, respectively. A significant association was found between complex fistulas, higher Parks grades, and the total length of the fistula tract.

**Conclusion:**

This study elucidates MRI patterns of perianal fistulas, revealing that over one-third of patients had complex fistulas. This underscores the importance of preoperative MRI for effective surgical planning and reducing recurrence rates.

## Introduction

Perianal fistulas are abnormal channels that extend from the anal canal to the perianal skin or perineum, with a prevalence in the general population estimated between 0.01% and 0.05% (1). These figures are likely understated due to patients' reluctance to seek medical help due to embarrassment (2). Magnetic Resonance Imaging (MRI) has transformed the imaging of perianal fistulas, providing high-resolution images that accurately depict the complex anatomy of the anal canal, enabling effective preoperative planning and reducing recurrence rates (3).

Perianal fistulas occur more frequently in men than women (4) and are often idiopathic (>90%), though they can also arise from underlying conditions like inflammatory bowel disease, anorectal cancer, tuberculosis, pelvic radiotherapy, or trauma (1,2,5-7). Typically, idiopathic fistulas originate from glandular crypts at the dentate line, influenced by inflammatory processes affecting the proctodeal glands.

Classification systems for perianal fistulas, notably the Parks classification, categorize them based on their relationship to the external sphincter (Table 1) (8, 9). The St. James University Hospital grading scheme also assists in radiological reporting (Table 2) (1, 10), addressing secondary extensions and complications like abscesses, which are significant for surgical planning (9).

**Table 1:** Parks classification of perianal fistula (31)

Type	Description
Intersphincteric	The tract doesn't cross the external sphincter
Transphincteric	The tract crosses the external sphincter to the ischioanal fossa
Suprasphincteric	there is penetration of the levator ani by the fistula tract
Extrasphincteric	Fistula tract penetrates the levator ani and opens into the rectum or sigmoid colon with no involvement of anal canal.

**Table 2:** St James's University Hospital Classification system of perianal fistulas (8)

Grade 1	Simple Linear Inter-sphincteric Fistula.
Grade 2	Intersphincteric Fistula with Abscess or Secondary Track.
Grade 3	Trans-sphincteric Fistula.
Grade 4	Trans-sphincteric Fistula with Abscess or Secondary Track within the Ischiorectal Fossa.
Grade 5	Supralevator and Translevator Disease.

Surgical intervention carries a high recurrence risk (>25%), necessitating detailed characterization of the fistula tract and the identification of any secondary extensions or abscesses (7). While various imaging modalities exist, MRI has emerged as the preferred technique, offering comprehensive visualization of anal canal anatomy(11-15).

Key MRI sequences, such as T2-weighted imaging with and without fat suppression, are essential for evaluating perianal fistulas (4). Diffusion-weighted imaging further enhances diagnostic accuracy by identifying active disease and abscess formations (16).

Despite the critical role of MRI in managing perianal fistulas, literature on this topic in Ethiopia is scarce. Observations indicate a shift from conventional fistulography to MRI, particularly in urban areas like Addis Ababa, although high costs and limited access impede widespread adoption. This study aims to enhance understanding of MRI patterns of perianal fistulas locally and assess complications that may influence surgical approaches and patient management.

## Materials and Methods

**Study area and design**: A cross-sectional study design was employed, utilizing retrospectively collected data from a private diagnostic center in Addis Ababa over one year, from May 2023 to June 2024. The center is equipped with a 1.5 Tesla GE MRI machine, which typically examines 3-5 patients with suspected perianal fistulas weekly. Exhaustive sampling included all eligible patients within the study period.

**Population**: The Picture Archiving and Communication System (PACS) system of the diagnostic center identified 276 patients with perianal fistulas during the study period. Inclusion criteria encompassed all patients who underwent pelvic MRI, ensuring standard parameters, including mandatory T2-weighted fat-suppressed sequences and diffusion-weighted imaging. Exclusion criteria included patients with prior perianal surgery that distorted anatomy or those lacking standard MRI protocols.

**Data collection procedure**: A structured questionnaire was developed to collect clinical data and MRI findings. The questionnaire underwent pretesting and refinement. MRI images were retrieved from the PACS system and independently reviewed by a body imaging fellow and subsequently by a body imaging subspecialist. Discrepancies were resolved through consensus discussions. Clinical and sociodemographic information were obtained from the PACS system.

No bowel preparation was performed before imaging, which took place on a 1.5 Tesla MR machine [General Electric (GE) Medical Systems] with a pelvic phased-array coil. Imaging sequences included T1-weighted, T2-weighted (sagittal and axial), and diffusion-weighted sequences, among others. The MRI scans were analyzed to identify the primary tract's orientation in relation to the anal clock, its course, and its relationship to the anal sphincter complex. The location of the internal fistula opening was determined based on axial images, with reference to the clock face. Secondary extensions, multiple tracts, and abscess collections were also assessed. The Parks and St. James classifications were used to categorize the perianal fistulas.

### Operational definitions

**Primary Fistula Tract**: An abnormal perianal T2 hyperintense tract with internal (anal) and external (skin) openings.

**Figure 1:**
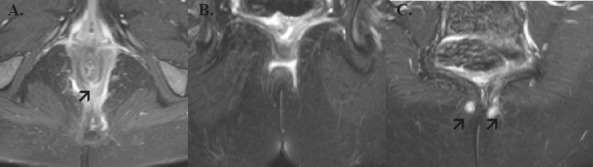
Axial sections of An MRI T2 Weighted image with fat suppression shows a horseshoe fistula tract with internal opening seen at 6 o'clock (shown by an arrow in A) considering the anal canal as a clock face. The tract crosses the midline (seen in B) and has two external openings to the right and left of the natal cleft (shown by the arrow in C)

**Horse-Shoe Tract**: A tract that appears on both sides of the internal opening or crosses the midline. (Figure 1)

**Complex Fistula**: Defined by the presence of secondary extensions, abscess collections, or additional perianal diseases.

**Figure 2:**
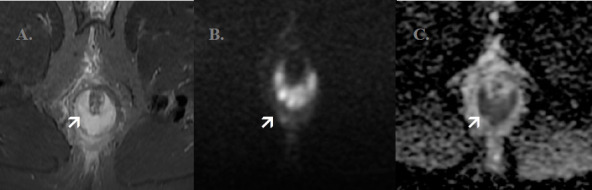
Axial sequence of MRI of a patient with perianal abscess: There is intersphincteric T2 hyperintense collection shown by the arrow in A which has diffusion restriction as shown by the arrow in images B and C

**Perianal Abscess**: A T2 hyperintense collection larger than 1 cm, exhibiting diffusion restriction. (Figure 2)

**Trans-Sphincteric Fistula**: A fistula or abscess that crosses the external sphincter (17).

**Supralevator Fistula**: A fistula or abscess that crosses the levator ani (17).

**Statistical analysis**: Statistical analysis was conducted using IBM SPSS version 27. Frequency tables and graphs described the study variables, and the chi-square test assessed associations, with P values < 0.05 deemed statistically significant.

**Ethical approval**: Ethical approval was obtained from the institutional review board. Informed consent was not required, as this was a retrospective review of imaging findings. The study adhered to the Helsinki Declaration, maintaining strict confidentiality for all clinical and radiological data.

## Results

**Study population characteristics**: A total of 279 patients with perianal fistulas were imaged over the past year and met the inclusion criteria. Among these, 233 (84.4%) were male and 46 (15.6%) were female, resulting in a male-to-female ratio of 5.4:1. No statistically significant differences were observed between genders regarding tract multiplicity, complex fistula features (including secondary extensions and abscess collections), fistula grades, or types.

Most patients (175, 63%) were aged 25-44, with a mean age of 38 years (±11). Of the total, 93 (33.7%) underwent surgical management. The most common clinical presentation was perianal discharge, noted in 217 (47.6%) cases, followed closely by perianal pain in 114 (25.0%) and swelling in 117 (25.7%). The mean duration of clinical symptoms was 22.7 months.

**Figure 3:**
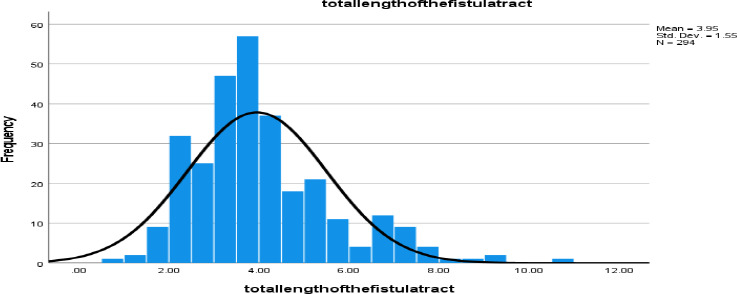
Histogram shows the distribution of the total length of a perianal fistula tracts identified in 276 patients from May 2023-Jun 2024

**Primary Fistula Tract**: In total, 304 primary fistula tracts were identified among 276 patients. Of these, 254 (83.6%) were single tracts, while 22 (7.2%) patients had multiple tracts, with a maximum of four per patient (Figure 3). Based on signal intensity, 293 (96.4%) were classified as active fistulas, while 4 (3.3%) were fibrosed. The average distance from the anal verge to the internal opening of the primary tract was 2.7 cm (±0.86 cm), and the mean total length of the primary tract was 3.95 cm (±1.5 cm).

**Figure 4:**
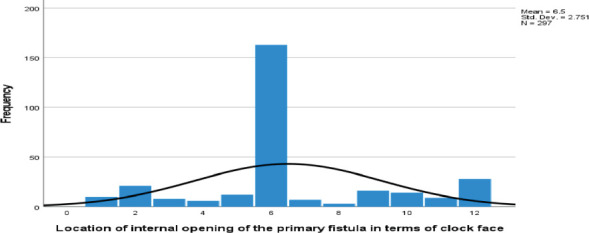
Location of internal opening of primary fistula in terms of the clock face in 304 fistula tracts from May 2023-Jun 2024

**Internal Opening**: The internal opening of the fistula tract was visualized in 299 (98.4%) cases. In 163 (53.6%) cases, the internal opening was located at the 6 o'clock position, followed by 28 (9.2%) at 12 o'clock and 21 (6.9%) at 2 o'clock (Figure 4).

**External Opening**: A total of 334 external openings were identified in 276 patients. A single external opening was present in 228 (82.6%) cases, while multiple openings were found in 39 (14.1%) cases with two openings, and 6 (2.2%) cases with three. A few patients had four or six external openings. The primary external openings were categorized into anterior midline, posterior midline, right, and left of the natal cleft, with the most common being the posterior midline (99 cases, 32.6%)(Table 3).

**Table 3:** Location of internal opening of primary fistula in terms of the clock face in 304 fistula tracts from May 2023-Jun 2024

Location of external opening	Number (n)	Valid percent
Anterior midline	37	12.3
Right of natal cleft	90	30.0
Posterior midline	99	33.0
Left of natal cleft	74	24.7
Total	300	100.0
Unidentified opening	4	

**Secondary Extensions and Abscess Collection**: Sixty-four (27.1%) patients exhibited secondary extensions, with some having up to three extensions, and one patient had four. The most common site for secondary extensions was the intersphincteric region (30 cases, 46.9%), followed by extrasphincteric locations (18 cases, 28.1%). Additionally, 43 (15.6%) patients had abscess collections, with a mean size of 2.3 cm (±1.2 cm). The most common site for abscess collections was intersphincteric (23 cases, 53.5%), followed by perianal (16 cases, 37.2%), with supralevator and transsphincteric abscesses in 3 (7%) and 1 (2.3%) cases, respectively.

**Grading and Classification**: Using the Parks and St. James classification systems, the most common type of fistula was intersphincteric, found in 238 cases (78.3%), followed by transsphincteric in 45 cases (14.8%). According to the St. James classification, 176 (57.9%) cases were grade I, followed by grade II in 62 (20.4%), and grade III in 26 (8.6%). There were 19 (6.3%) grade IV and 16 (5.3%) grade V cases, which were the least common (Table 4).

**Table 4:** Parks and St. James classification of perianal fistula in 304 primary tracts identified in 276 patients from May 2023-Jun 2024

Parks classification	Number(n)	Percent (%)	St. James classification	Number(n)	Percent (%)
Intersphincteric	238	78.3	grade 1 grade 2	176 62	57.9 20.4
Transsphincteric	45	14.8	grade 3 grade 4	26 19	8.6 6.3
Suprasphincteric	14	4.6	grade 5	16	5.3
Extrasphincteric	2	0.7			
Unclassified	5	1.6	Unclassified	5	1.6
Total	304	100.0	Total	304	100.0

**Correlation Studies**: Correlation analysis revealed a significant association between higher Parks grades and the presence of complex fistulas (Pearson correlation of 0.288*, p < 0.001). A statistically significant correlation was also found between complex fistulas and the total length of the fistula tract (Pearson correlation = 0.318, p < 0.001) (Table 5). No significant correlations were identified for other examined variables, including the presence of abscesses and different grades of perianal fistulas.

**Table 5:** Correlation between complex fistula and other variables in 304 primary tracts identified in 276 patients from May 2023-Jun 2024

Variable	Pearson Correlation	Significance (p-value)
**Previous Surgery**	0.288*	**<0.001** **
**Total length of the fistula tract**	**0.318**	**<0.001** **
**Sex**	0.013	0.855
**Perianal pain**	-0.059	0.356
**Perianal mass**	-0.046	0.482
**Perianal discharge**	0.097	0.195
**Perianal swelling**	0.046	0.483
**Age**	0.058	0.349

** has significant association with P-value <0.05%

## Discussion

The literature on perianal fistulas in Ethiopia and tropical Africa is limited, yet findings suggest a significant prevalence. A study on colorectal and perianal surgery in Ethiopia found that benign perianal conditions accounted for over 50% of cases, with perianal fistulas representing 43.4% of these disorders (18). This underexplored area highlights the need for further research.

Globally, the value of MRI in assessing perianal fistulas has been well documented (19,20). A randomized trial indicated that preoperative MRI led to an 88% operative success rate, compared to 44% for those undergoing surgery without prior imaging (21).

In this study, 233 (84.4%) of the 279 patients were male, yielding a male-to-female ratio of 5.4:1. This ratio is slightly lower than that in previous Ethiopian studies (18) but aligns with findings from Nigeria (22) and Saudi Arabia (19), where male predominance is observed. The reasons behind this gender disparity remain unclear, although some hypotheses suggest hormonal factors or anatomical differences (23).

The age distribution revealed that 63% of patients were between 25-44 years, with a mean age of 38 years. This finding parallels other studies (18, 22, 24), indicating that perianal conditions predominantly affect early to middle adulthood.

Identifying whether a fistula tract is active or fibrotic is important as it helps avoid unnecessary exploration (21) The vast majority of the fistula tracts in this study were labeled as active, accounting for 293 (96.4%) of the cases, while the remaining 4 (3.3%) were considered fibrosed. The loss of hyperintense signal on T2-weighted imaging (T2WI) is the initial imaging manifestation of a healing tract, followed by an absence of contrast enhancement (14).

Distance of internal opening of primary tract from anal verge measured on the coronal plane was on average 2.7cm ± 0.86 cm and the mean total length of the primary tract was 3.95cm ± 1.5cm. This finding supports the observation that the internal opening of a perianal fistula is usually situated at the level of the dentate line (4). An MRI based study in India found most of the fistula having total length in the range of 2-6cm which is similar to this study with 3-5cm being the commonest range (25). There was strong correlation between complex fistula and the total length of the fistula tract. This could be because complex fistulas tend to have secondary extensions which may increase the overall length of the tract (26). Another reason can be the repeated chronic infection in perianal fistula resulting in longer tract formation which is difficult to treat (27).

The predominant location of internal openings at the 6 o'clock position was consistent with existing literature. Secondary extensions and abscess collections were also notable, with intersphincteric locations being the most common. These factors are critical for preoperative planning and minimizing recurrence.

According to the Parks classification, intersphincteric fistulas were most common, followed by transsphincteric types. The findings correspond with previous research in Saudi Arabia, Turkey, and India (7, 19, 28). Higher-grade fistulas were more likely to be complex, indicating that they traverse deeper tissue layers, increasing infection risk and the potential for secondary extensions (26). This is also supported by an international survey done on the contemporary management of perianal fistula (29).

This study was conducted in an urban setting, where MRI access is more readily available. Access may be limited in rural areas due to cost and availability, highlighting the need for future research on MRI accessibility in resource-limited settings.

In conclusion, the intersphincteric type of perianal fistula and St. James grades I and II were most common in this study. The presence of complex fistulas, abscesses, and secondary extensions underscores the importance of preoperative MRI, as unidentified features contribute to recurrence. Collaborative efforts among surgeons and radiologists are vital for successful management (3, 30).

Limitations include the retrospective design and single-center focus. Future research should involve multi-center, large-scale prospective studies to enhance understanding and management of perianal fistulas in Ethiopia. Additionally, exploring advanced imaging techniques and long-term outcomes will be crucial for integrating MRI into clinical practice.
